# Effects of Taurine Depletion on Body Weight and Mouse Behavior during Development

**DOI:** 10.3390/metabo12070631

**Published:** 2022-07-09

**Authors:** Miho Watanabe, Takashi Ito, Atsuo Fukuda

**Affiliations:** 1 Department of Neurophysiology, Hamamatsu University School of Medicine, Hamamatsu 431-3192, Japan; mihow@hama-med.ac.jp; 2 Department of Bioscience and Technology, Graduate School of Bioscience and Technology, Fukui Prefectural University, Fukui 910-1195, Japan; tito@fpu.ac.jp

**Keywords:** taurine, taurine transporter, knockout mice, brain, skeletal muscle, body weight, behavior

## Abstract

Taurine (2-aminoethanesulfonic acid) plays an important role in various physiological functions and is abundant in the brain and skeletal muscle. Extracellular taurine is an endogenous agonist of gamma-aminobutyric acid type A and glycine receptors. Taurine actively accumulates in cells via the taurine transporter (TauT). Adult taurine-knockout (*TauT*^−/−^) mice exhibit lower body weights and exercise intolerance. To further examine the physiological role of taurine, we examined the effect of its depletion on mouse behavior, startle responses, muscular endurance, and body weight during development from postnatal day 0 (P0) until P60. In the elevated plus maze test, *TauT*^−/−^ mice showed decreased anxiety-like behavior. In addition, *TauT*^−/−^ mice did not show a startle response to startle stimuli, suggesting they have difficulty hearing. Wire-hang test revealed that muscular endurance was reduced in *TauT*^−/−^ mice. Although a reduction of body weight was observed in *TauT*^−/−^ mice during the developmental period, changes in body weight during 60% food restriction were similar to wild-type mice. Collectively, these results suggest that taurine has important roles in anxiety-like behavior, hearing, muscular endurance, and maintenance of body weight.

## 1. Introduction

Taurine (2-aminoethanesulfonic acid) is one of the most ubiquitous and abundant amino acids in mammalian tissues [1,2], whereby it plays important roles in various physiological functions [3] such as mitochondrial translation [4], Ca^2+^ homeostasis [5], cell volume regulation [6], stability of membranes [7], and antioxidant effects [8]. Although the capacity to synthesize taurine is limited in most tissues [9], high levels of intracellular taurine are maintained by the taurine transporter (TauT/SLC6a6) on the membrane [10]. TauT is a Na^+^-Cl^−^ ion-dependent transporter expressed ubiquitously in mammalian tissues [10]. The development of TauT-knockout (*TauT*^−/−^) mice by two laboratories revealed the physiological functions of taurine [11,12]. *TauT*^−/−^ mice exhibit shortened lifespan; cardiomyopathy; aging-dependent cardiac dysfunction; hepatitis; and renal, visual, auditory, and olfactory dysfunction [13,14,15]. Transcriptome microarray analysis of *TauT*^−/−^ mice revealed that pro-fibrotic genes, such as S100 calcium-binding protein A4 (S100A4), actin alpha 2, smooth muscle (ACTA2), and connective tissue growth factor (CTGF), were increased in *TauT*^−/−^ mice hearts [16]. Based on the transcriptome-pathway analysis, the genes involved in the “organization of extracellular matrix,” such as galectin 3 (LGALS3), are enriched in old *TauT*^−/−^ mice hearts, suggesting the contribution of these genes to fibrosis [16].

Taurine is present in high concentrations in the brain [17,18,19]. Moreover, taurine levels are high in the immature brain compared with the adult brain [18]. Taurine is required for the development of the central nervous system [20,21,22]. Our prior reports demonstrated that ambient taurine tonically activates gamma-aminobutyric acid type A (GABA_A_) receptors and slows radial migration in the developing cerebral cortex [23]. In addition, taurine regulates the temporal specification of excitatory glutamatergic neuronal progenitors in the developing cortex [24]. In immature neurons, taurine inhibits the K^+^-Cl^−^ cotransporter KCC2, which maintains low intracellular Cl^−^ levels critical for mediating fast-hyperpolarizing synaptic inhibition by GABA_A_ and glycine receptors via the with-no-lysine (WNK) protein kinase signaling pathway [25]. However, the effects of taurine depletion on adult mouse behaviors, such as locomotor activity, anxiety-like behavior, and startle response, have not yet been reported.

Taurine also occurs in particularly high concentrations in skeletal muscle [11]. *TauT*^−/−^ mice show a more than 98% reduction in muscle taurine content [12,26]. In addition, *TauT*^−/−^ mice exhibit exercise intolerance in both treadmills and forced swimming tests [26,27], as well as muscular structural defects, such as myofibril derangement and the appearance of autophagic bodies [12,14]. *TauT*^−/−^ mice exhibit accelerated skeletal muscle aging and growth, and differentiation factor 15 (GDF15), which is a member of the transforming growth factor-β superfamily, was elevated during aging in *TauT*^−/−^ mice [28]. A previous report found that adult *TauT*^−/−^ mice fed a normal diet had lower body weights and visceral fat, although the body weights of *TauT*^−/−^ mice fed a diet containing 60% fat increased to a level comparable to that of wild-type (WT) mice [29]. *TauT*^−/−^ mice showed lower fasting blood glucose and were more tolerant against glucose injection [29]. Metabolism of fatty acids was suppressed by the downregulation of genes and enzymes involved in fatty acids β-oxidation [27]. Taurine reduced obesity in WT mice fed a high-fat diet, suppressed increases in adipocyte size, body fat, and body weight [30] and improved insulin sensitivity, and increased energy expenditure and adaptive thermogenesis by elevating the browning of white adipose tissue [31]. Body weight and height of infants at birth were significantly higher in the high taurine intake pregnant women compared to infants in low taurine intake pregnant women [32]. However, changes in body weight of *TauT*^−/−^ mice during development remain unclear.

In this study, to further examine the physiological role of taurine, we used *TauT*^−/−^ mice to investigate the effects of taurine deficiency on body weight changes during development and in adult mice during food restriction. In addition, we measured muscular endurance. Furthermore, to examine the effects of taurine deficiency on behavior, we performed several behavioral analyses.

## 2. Results

### 2.1. TauT^−/−^ Mice Showed Decreased Anxiety-Like Behavior

**Figure 1:**
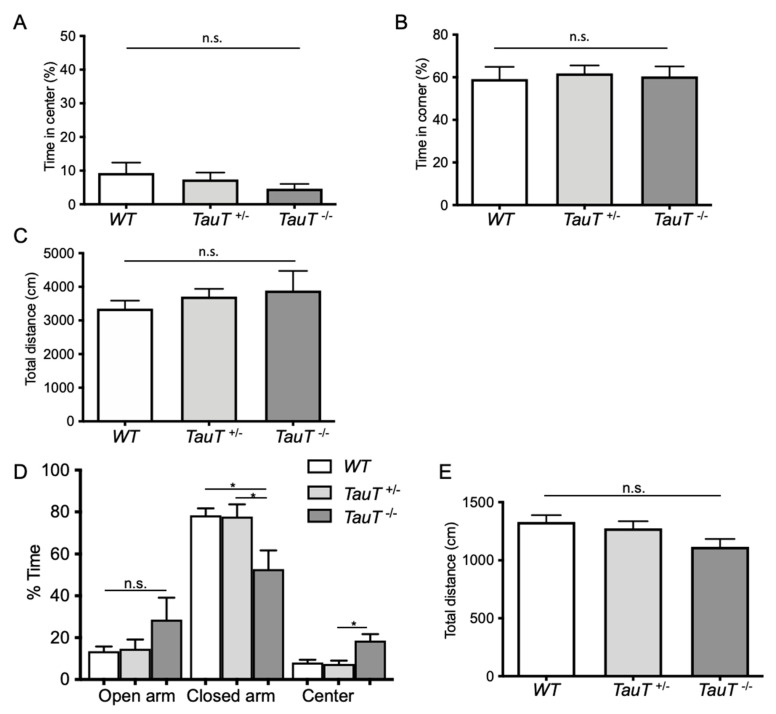
(**A**–**C**) In the open field test, mice were placed in the center of the open field apparatus and their movement was tracked for 10 min. The percentage of time spent in the center (**A**), percentage of time spent in the corner (**B**), and total distance moved in an open field (**C**) were measured for WT, *TauT*^+/−^, and *TauT*^−/−^ mice. (**D**,**E**) In the elevated plus maze test, mice were placed in the center of the elevated plus maze and allowed to explore for 5 min. The percentage of time spent in the open arms, closed arms, and center (* *p* < 0.05, n.s. = not significant by Kruskal–Wallis test) (**D**) and total distance moved (**E**) in the elevated plus maze were measured. Error bars represent S.E.M.

We performed a behavioral analysis of *TauT*^−/−^ mice. To assay general locomotor activity levels and anxiety, we performed an open field test. Percentages of time spent in the center (WT, 9.3 ± 3.1%, *n* = 12; *TauT*^+/−^, 7.4 ± 2.0%, *n* = 12; *TauT*^−/−^, 4.7 ± 1.4%, *n* = 8) and time spent in the corner (WT, 59.2 ± 5.7%; *TauT*^+/−^, 61.9 ± 3.7%; *TauT*^−/−^, 60.4 ± 4.7%) were similar between genotypes (Figure 1A,B). Moreover, no significant difference in the total distance moved was found between genotypes (WT, 3354.1 ± 234.1 cm; *TauT*^+/−^, 3690.5 ± 214.9 cm; *TauT*^−/−^ mice, 3891.4 ± 582.9 cm; Figure 1C). From these results, *TauT*^+/−^ and *TauT*^−/−^ mice both showed normal locomotor activity. To examine anxiety-like behavior, we performed an elevated plus maze test. The percentage of time spent in the open arm was increased in *TauT*^−/−^ mice compared with WT and *TauT*^+/−^ mice, although there were no significant differences between groups (WT, 13.5 ± 2.3%, *n* = 13; *TauT*^+/−^, 14.7 ± 4.4%, *n* = 11; *TauT*^−/−^, 28.6 ± 10.5%, *n* = 11; Figure 1D). The percentage of time spent in the closed arm was significantly decreased in *TauT*^−/−^ mice compared with WT and *TauT*^+/−^ mice (WT, 78.4 ± 3.3%; *TauT*^+/−^, 77.8 ± 5.8%; *TauT*^−/−^, 52.8 ± 9.0%). In addition, the percentage of time spent in the center was significantly increased in *TauT*^−/−^ mice compared with WT mice and showed an increased trend compared with WT mice (*p* = 0.06; WT, 8.1 ± 1.4%; *TauT*^+/−^, 7.4 ± 1.6%; *TauT*^−/−^, 18.6 ± 3.1%). No significant difference in total distance moved was observed between genotypes (WT, 1329.7 ± 58.2 cm; *TauT*^+/−^, 1274.9 ± 61.1 cm; *TauT*^−/−^, 1117.1 ± 67.0 cm; Figure 1E). These results suggest that *TauT*^−/−^ mice showed decreased anxiety-like behavior and had difficulty making decisions underlying approach/avoidance conflict and risk assessment.

### 2.2. TauT^−/−^ Mice Showed Difficulty Hearing

Next, we performed the startle response test and checked the dependence of startle responses on startle stimulus intensity (60 to 120-dB pulse, WT *n* = 6, *TauT*^−/−^ *n* = 6). WT mice showed a startle response to startle pulses between 90 and 120 dB (Table 1). In total, 50% of WT mice showed a startle response to a startle pulse of 90 dB. All WT mice showed startle responses to startle pulses between 100 and 120 dB. However, no *TauT*^−/−^ mice exhibited a startle response to any startle pulse. From these results, *TauT*^−/−^ mice likely have difficulty hearing.

### 2.3. Muscular Endurance Was Reduced in TauT^−/−^ Mice

**Figure 2:**
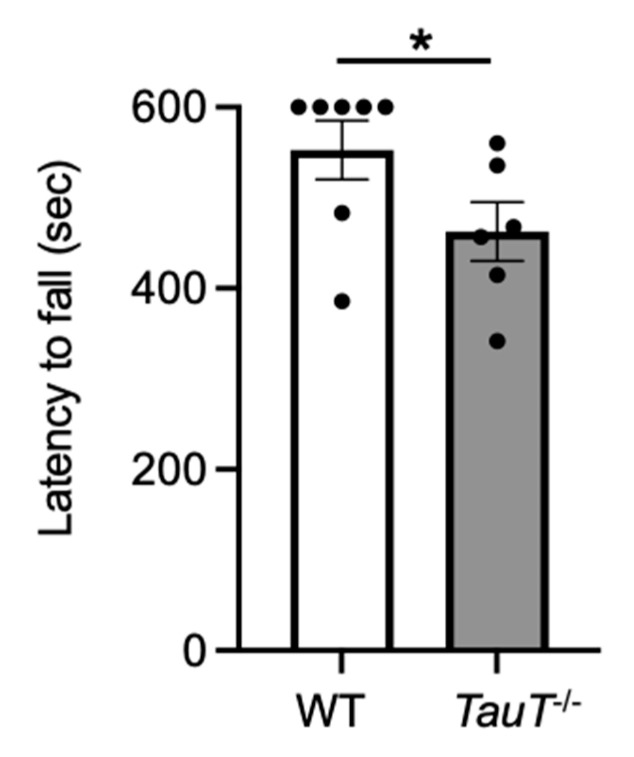
Muscular endurance was reduced in *TauT*^−/−^ mice. Wire-hang test was performed. Average latency to fall from the wire mesh of two trials in WT and *TauT*^−/−^ mice. * *p* < 0.05 by Mann-Whitney test. Error bars represent S.E.M. Closed circles represent animals.

A previous report suggested that exercise capacity (as determined by a treadmill test) was reduced in *TauT*^−/−^ mice [27]. Therefore, to measure muscular endurance, a wire-hang test was performed. Each mouse was placed on a wire mesh and then turned upside down. The latency to fall was measured. Latency to fall was reduced in *TauT*^−/−^ mice compared with WT mice (WT, 552.6 ± 32.4 s, *n* = 7; *TauT*^−/−^, 462.6 ± 32.6 s, *n* = 6; Figure 2). These results suggest that muscular endurance was reduced in *TauT*^−/−^ mice.

### 2.4. Body Weight Gain Was Reduced in TauT^−/−^ Mice during Development

**Figure 3:**
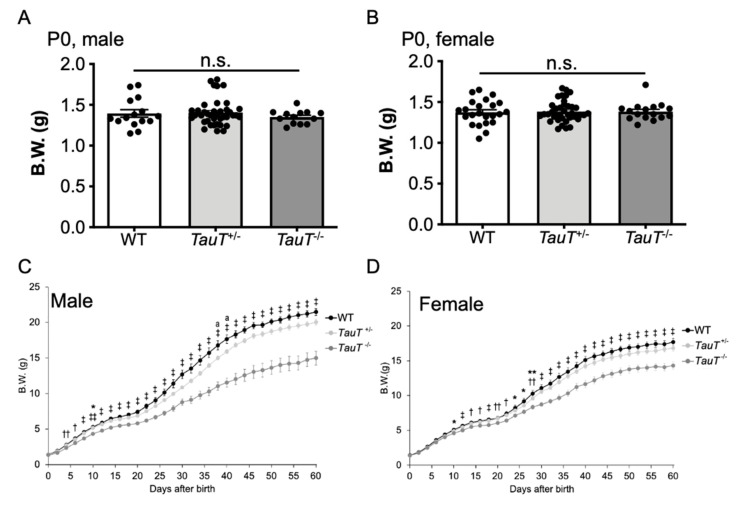
*TauT*^−/−^ mice exhibited lower body weights during development. (**A**) Body weights of male WT, *TauT*^+/−^, and *TauT*^−/−^ pups at P0. Closed circles represent animals. (**B**) Body weights of female WT, *TauT*^+/−^, and *TauT*^−/−^ pups at P0. (**C**) Changes in body weights of male WT, *TauT*^+/−^, and *TauT*^−/−^ mice during development. (**D**) Changes in body weights of female WT, *TauT*^+/−^, and *TauT*^−/−^ mice during development. Error bars represent S.E.M. * *p* < 0.05 *TauT*^−/−^ vs. WT, ** *p* < 0.01 *TauT*^−/−^ vs. WT, † *p* < 0.05 *TauT*^−/−^ vs. WT and *TauT*^+/−^, ‡ *p* < 0.01 *TauT*^−/−^ vs. WT and *TauT*^+/−^, †† *p* < 0.05 *TauT*^−/−^ vs. *TauT*^+/−^, ‡‡ *p* < 0.01 *TauT*^−/−^ vs. *TauT*^+/−^, and ^a^ *p* < 0.05 *TauT*^+/−^ vs. WT by Kruskal-Wallis test.

Adult *TauT*^−/−^ mice showed reduced body weights [29], so we examined changes in body weight during development from postnatal day 0 (P0) until P60. The body weight of each mouse was measured every 2 days. At birth, there were no differences in body weights between genotypes for either male (WT, 1.39 ± 0.05 g, *n* = 15; *TauT*^+/−^, 1.41 ± 0.03 g, *n* = 39; *TauT*^−/−^, 1.35 ± 0.02 g, *n* = 13) or female (WT, 1.37 ± 0.03 g, *n* = 23; *TauT*^+/−^, 1.38 ± 0.02 g, *n* = 39; *TauT*^−/−^, 1.38 ± 0.03 g, *n* = 16) mice (Figure 3A,B). However, body weights of male *TauT*^−/−^ mice were significantly lower compared with male WT and *TauT*^+/−^ mice between P4 onward (WT, *n* = 12; *TauT*^+/−^, *n* = 32; *TauT*^−/−^, *n* = 12) (Figure 3C). In addition, a reduction in body weights of female *TauT*^−/−^ mice was observed between P10 onward (WT, *n* = 16; *TauT*^+/−^, *n* = 32; *TauT*^−/−^, *n* = 15) (Figure 3D). The difference in body weight seems to become apparent around P20 when mice start self-feeding. Notably, the difference in body weights between WT and *TauT*^−/−^ mice was greater in male mice than in female mice.

### 2.5. Body Weight Changes during 60% Food Restriction Were Similar in WT and TauT^−/−^ Mice

**Figure 4:**
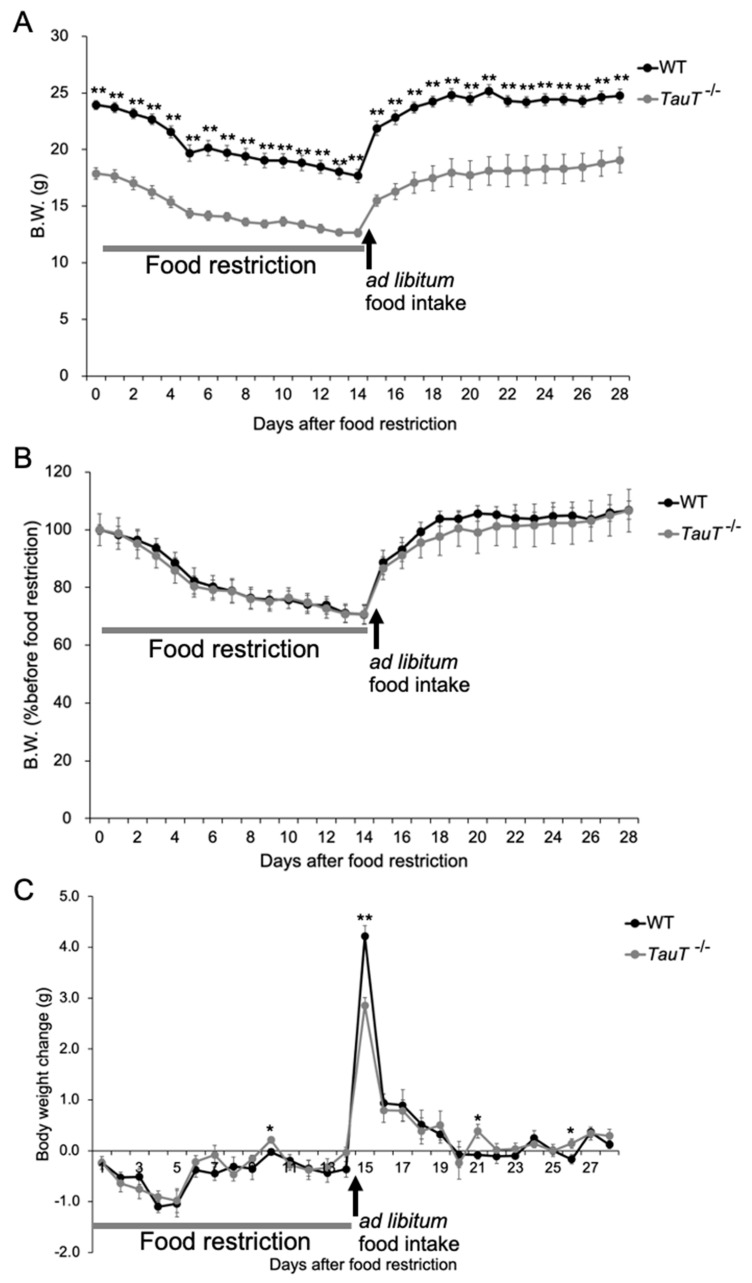
Changes in body weights following 60% food restriction in WT and *TauT*^−/−^ mice. (**A**) Changes in body weight after 60% food restriction and recovery (food *ad libitum*) in WT mice and *TauT*^−/−^ mice. (**B**) Body weight changes are shown as a percentage of the initial body weight before 60% food restriction. (**C**) Body weight loss or gain after 60% food restriction and recovery (food *ad libitum*) in WT mice and *TauT*^−/−^ mice. Error bars represent S.E.M. * *p* < 0.05, ** *p* < 0.01 by Kruskal-Wallis test.

A previous report showed that the weight of visceral fat was significantly less in *TauT*^−/−^ mice compared with WT mice; however, when fed a high-fat diet, *TauT*^−/−^ mice exhibited body weights comparable to WT mice and dramatically increased abdominal fat after 16 weeks [29]. Therefore, we examined the effect of a 60% food restriction. Mice were given a food pellet equal to 60% of daily food intake before the onset of the dark phase for 2 weeks. Subsequently, mice were given food *ad libitum* for 2 weeks. Body weights were measured every day. Body weights were markedly decreased during the first 5 days after starting 60% food restriction, then gradually decreased in both WT and *TauT*^−/−^ mice (WT, *n* = 7; *TauT*^−/−^, *n* = 7; Figure 4A,B). After WT and *TauT*^−/−^ mice were given food *ad libitum*; their body weights increased in both WT and *TauT*^−/−^ mice. Indeed, the remarkable weight loss observed during the first 5 days after starting 60% food restriction gradually recovered in both WT and *TauT*^−/−^ mice (Figure 4C). On the first day, mice were given food *ad libitum*; the weight gain was significantly lower in *TauT*^−/−^ mice (2.9 ± 0.1 g) compared with WT mice (4.2 ± 0.2 g). Thus, *TauT*^−/−^ mice showed an apparent disturbance of body weight recovery after food restriction compared with WT mice, although their body weight reduction during food restriction was comparable.

### 2.6. Comprehensive Analysis of Protein Kinases and Target Proteins

Metabolic processes are commonly regulated by a series of phosphorylation events by kinases. Therefore, we performed an active site-directed competition binding assay to quantitatively measure interactions between kinases and target proteins to compare differences between WT and *TauT*^−/−^ mice. Nineteen proteins showed significant changes in expression levels between WT and *TauT*^−/−^ mice. Moreover, three proteins exhibited significant changes in the phosphorylation of analyzed sites (Table S1). The most prominent protein alteration was phosphorylation of signal transducer and activator of transcription 3 (STAT3) at tyrosine 705, which was increased by 9449% in the brains of *TauT*^−/−^ mice compared with WT mice.

## 3. Discussion

We have shown that taurine deficiency in mice decreased anxiety-like behaviors, abolished startle responses, and reduced muscular endurance. Furthermore, taurine deficiency caused lower body weight gain during development. Our findings demonstrate the importance of taurine in brain function, muscular endurance, and energy metabolism.

Our results show that *TauT*^−/−^ mice exhibited decreased anxiety-like behaviors, as indicated by reduced time in the closed arm of the elevated plus maze test and increased time spent in the center square. Time spent exploring the center square has been suggested to reflect decision-making underlying approach-avoidance conflict and risk assessment. Although *TauT*^−/−^ mice exhibit visual dysfunction due to retinal degeneration [11] and hearing loss [13], the elevated plus maze test is based on mouse exploratory patterns that avoid open spaces and are motivated by thigmotaxis; the tendency to be close to vertical surfaces is far more reliant on tactile inputs compared with vision [33]. Therefore, we consider that visual dysfunction and hearing loss have little effect on our behavioral tests. Taurine is present at high concentrations in the brain [11], whereby it acts as a partial agonist of GABA_A_ [34] and glycine receptors [35]. Although the physiological roles of taurine in the brain are still not fully understood, taurine is required for normal nervous system development [22]. In the developing brain, the taurine concentration is 3–4 times higher than present in the adult brain [18]. After the first postnatal week, taurine concentrations are downregulated [18]. Kittens from taurine-deficient mothers exhibit smaller brain weights and abnormal morphologies in the visual cortex [36] and cerebellum [37]. Ambient taurine in the mouse developing fetal cerebral cortex tonically activates GABA_A_ receptors and slows radial migration [23]. In addition, taurine increases the proliferation of neural stem cells [24,38]. Taurine modulates the switch of GABA responses from depolarizing to hyperpolarizing by suppressing KCC2 in the embryonic brain via WNK kinase signaling [25]. In the adult brain, taurine also activates extrasynaptic GABA_A_ receptors in the thalamus [39]. *TauT*^−/−^ mice show impaired GABAergic inhibition in the striatum [40]. Therefore, taurine deficiency in the brain may cause structural deficits or an imbalance in inhibitory and excitatory neurotransmission in neuronal circuits controlling anxiety behavior, thereby causing abnormal behavior in *TauT*^−/−^ mice.

*TauT*^−/−^ mice did not show startle responses to startle stimuli. Taurine is present at high concentrations in the organ of Corti [41] and inferior colliculus [42]. Since taurine concentrations are relatively high in glia [43], *TauT* knockout in glia cells may exacerbate degeneration of auditory nerve fibers, resulting in hearing loss [13]. Six-month-old *TauT*^−/−^ mice reportedly show 20-dB higher auditory brainstem response thresholds due to accelerated losses of outer and inner hair cells [13,44]. Taurine reduces neuronal activity via glycine receptors in the rat inferior colliculus [45]. In addition, taurine serves as a neuromodulator to enhance GABAergic and glycinergic transmission in the rat anteroventral cochlear nucleus [46]. Therefore, taurine may regulate neurotransmission of the central auditory system and its deficiency appears to cause difficulty hearing. The auditory brainstem response should be measured to precisely show *TauT*^−/−^ mice exhibit hearing loss. Further experiment is needed at this point.

*TauT*^−/−^ mice exhibited reduced body weights during the developmental period, although no difference was observed at birth. *TauT*^−/−^ mice reportedly show lower body weights and less abdominal fat at 3 months, although both food and water intake were unchanged [12,29]. *TauT*^−/−^ mice also exhibit lower weights of heart and tibial anterior muscle [12], as well as lower fasting blood glucose levels and tolerance against glucose injection [29]. Skeletal muscle lactate content is higher in *TauT*^−/−^ mice than in WT mice [15], suggesting that the glycolytic pathway may be accelerated in *TauT*^−/−^ mice. Acceleration of glycolysis can enhance glucose disposal from blood. In addition, several genes involved in the oxidation of fatty acids are downregulated in *TauT*^−/−^ mice [15], suggesting that their fatty acid utilization is reduced. Accordingly, glucose disposal may be enhanced in *TauT*^−/−^ mice as a compensatory mechanism. Therefore, imbalances in energy production and expenditure likely cause a reduction in body weight during development. Plasma insulin levels were lower in *TauT*^−/−^ mice compared with WT mice due to decreased numbers of beta cells in the pancreas [29]. Insulin promotes the conversion of glucose into fat. Therefore, lower insulin levels may reduce the conversion of glucose into fat, which may cause reductions in fat and body weight in *TauT*^−/−^ mice during development. In our comprehensive analysis of protein kinases and target proteins, STAT3 phosphorylation was significantly increased in *TauT*^−/−^ mouse brains. Leptin, a hormone synthesized and released from adipose tissue, plays a central role in the long-term control of body weight, acting mainly in the central nervous system to induce satiety, energy expenditure, and glycolysis through the STAT3 pathway [47,48]. Leptin binding to the long isoform of leptin receptor (LepRb) results in its dimerization, leading to the formation of the LepRb/Janus kinase 2 (JAK2) complex. The activated JAK2 phosphorylates itself and also Tyr985, Tyr1077, and Tyr1138 in LepRb. STAT3 binds to phospho-Tyr1077 in LepRb and are subsequently phosphorylated. Active STAT3 dimers then translocate to the nucleus and activate the transcription of their target genes, which mediate leptin’s anorexigenic effect. Therefore, there is a possibility that enhanced leptin signaling causes body weight and fat reduction in *TauT*^−/−^ mice during development. Taurine depletion did not affect body weight loss during 60% food restriction. Significant body weight reduction in *TauT*^−/−^ mice compared to WT mice was still observed during the food restriction and recovery period. *TauT*^−/−^ mice exhibited a significant reduction in body weight gain compared to WT mice on the first day of *ad libitum* food after food restriction. Phosphorylated STAT3 activates anorexigenic neurons that express proopiomelanocortin while inhibiting orexigenic neurons that express Agouti-related protein, thereby inhibiting food intake and increasing energy expenditure [47]. Combined with a previous report of *TauT*^−/−^ mice showing reduced abdominal fat weights [29], the present results show that extremely high levels of STAT3 phosphorylation may activate leptin’s anorexigenic effect, thereby reducing food intake in *TauT*^−/−^ mice even on the first day of *ad libitum* food after food restriction and disturb body weight recovery. The difference in body weights between WT and *TauT*^−/−^ mice was greater in male mice compared with female mice. The mechanism of these sex differences is unclear, but a previous paper showed that taurine supplementation increased the level of testosterone in male rats [49], that increased skeletal muscle. Therefore, there is a possibility that taurine depletion may reduce testosterone levels, which leads to a reduction of muscle mass, and results in body weight loss, especially in male mice. Further studies are needed to show the sex differences in weight regulation by taurine.

Taurine contents in skeletal muscle are high [11,12]. *TauT*^−/−^ mice reportedly show reduced swimming endurance times [12], as well as decreased maximum running speed and endurance duration in treadmill running tests [26,27], suggesting they exhibit exercise intolerance. Notably, taurine supplementation prolonged the time to exhaustion during treadmill running [50]. *TauT*^−/−^ mice show decreased myofiber size and myofiber disruption [12]. We previously performed LC-MS-based metabolome analysis and suggested that organic osmolytes such as betaine, glycerophosphocholine, and amino acids were increased in the heart muscle of *TauT*^−/−^ mice by compensating mechanisms for taurine depletion [51], although the expression of related gene expressions, such as betaine/GABA transporter-1 (BGT-1; Slc6a12), carnitine transporter Slc22a4 and Slc22a5 are not changed [52]. The mRNA expressions of heat shock protein 700 (Hsp70), the amino acid transporters (Slc38a2; ATA2), DnaJ (Hsp40) homolog, subfamily B, member 1 (Dnajb1), tumor necrosis factor receptor superfamily, member 12a (Tnfrsf12a), and S100 calcium-binding proteins (S100A4, S100A9), which are upregulated by osmotic stress, are elevated in both heart and skeletal muscle of the *TauT*^−/−^ mice [15], suggesting that dysfunction of osmoregulatory system impairs the tibial anterior muscle volume regulation. Taurine depletion reduced muscular endurance in this study, supporting previous findings of an important role for taurine in the maintenance of skeletal muscle function. Furthermore, STAT3 signaling plays important roles in regulating skeletal muscle mass, repair, and diseases [53]. Chronic activation of the interleukin 6/JAK/STAT3 signaling pathway is involved in muscle wasting. Activated STAT3 promotes skeletal muscle atrophy in muscle diseases, such as muscular dystrophy, cancer, and sepsis [54,55]. Therefore, very high levels of STAT3 phosphorylation may cause skeletal muscle abnormalities, thereby decreasing muscle endurance in *TauT*^−/−^ mice.

The limitation of this study is that we used whole-body *TauT* knockout mice. Since taurine is ubiquitous and is abundant free amino acid in the heart, retina, skeletal muscle, brain, and leukocytes [1,2], whole body *TauT* knockout causes various effects. Therefore, to demonstrate the functional role of taurine in specific tissue precisely, we need to generate tissue-specific *TauT* knockout mice. If we knockout *TauT* in a specific region of the brain, we can demonstrate the role of taurine in the behavior, excluding the effects of vision dysfunction and hearing loss. If we generate the time-specific *TauT* knockout mice, we can demonstrate the role of taurine at specific time points during development and in the adult mice. Further studies are needed to determine the precise role of taurine using cell/tissue- and time-specific *TauT* knockout mice. We have shown the effects of taurine depletion on anxiety-like behavior, startle response, muscular endurance, and body weight during development. However, the detailed mechanism by which taurine depletion causes such effects remains unclear. We have shown that *TauT*^−/−^ mice did not show startle responses. This result suggests that *TauT*^−/−^ mice might have hearing difficulty. However, we need to perform the auditory brainstem response test to confirm hearing difficulty in *TauT*^−/−^ mice and to locate hearing deficits along the auditory nerve pathway. Because *TauT*^−/−^ mice showed degeneration in the inner ear and auditory nerve, it is speculated that taurine has a role in the regulating survival of cells related to auditory function. Since taurine also acts as a partial agonist of GABA_A_ and glycine receptors, taurine might have a role in modulating the neurotransmission in the auditory nerve tract. Further studies are needed to show the underlying mechanisms of our results.

In conclusion, the present study suggests that taurine plays important roles in the maintenance of mouse behavior, hearing, body weight, and skeletal muscle function.

## 4. Materials and Methods

### 4.1. Animals

TauT-KO and littermate mice (C57BL/6 background) were used. The generation of TauT KO-mice was previously described [12]. Heterozygous TauT-KO male and female mice were mated, and homozygous TauT-KO (*TauT*^−/−^, male *n* = 51, female *n* = 17), heterozygous TauT-KO (*TauT*^+/−^, male *n* = 62, female *n* = 39), and wild-type (WT, male *n* = 60, female *n* = 24) mice were used for experiments. Genotyping for TauT-KO mice was carried out by PCR of mouse tail DNA using the following primers: 5′-GGTGTCACTAAGACGTGAGTTG-3′, 5′-CAGACAGCACAAAGTCGATCT-3′, and 5′-TGCTAAAGCGCATGCTCCAGACTG-3′. Mice were kept in a 12-h light/dark cycle (lights on at 07:00 a.m.) with access to food and water *ad libitum* unless otherwise noted. All experimental procedures were approved by the institutional animal care and use committee of Hamamatsu University School of Medicine. With regard to the ethical use of animals for experimentation, all efforts were made to minimize the number of animals used and their suffering.

### 4.2. Measurements of Body Weight and Food Restriction

Body weights of male and female mice aged P0–P60 were measured once every 2 days between 16:00 and 19:00 h. For the food-restriction experiment, daily food consumption of WT mice was measured for 10 days and average food consumption per gram of body weight was calculated. After 7 days of individual housing for acclimation, mice were given a food pellet equal to 60% of their average daily food intake. Body weights were measured between 16:00 and 19:00 h for 14 days during 60% food restriction. After food restriction, mice were allowed free access to food, and body weights were measured for an additional 14 days to observe recovery.

### 4.3. Behavioral Analysis

For the open field test, the size of the open field box was 42 × 42 × 40 cm. Mice were placed in the center of the chamber at the beginning of each test. The movement of each mouse was tracked for 10 min. Locomotor activity was recorded and analyzed using a video tracking system (SMART 3.0; Panlab, Cornellà de Llobregat, Spain). For the elevated plus maze test, we used an elevated plus maze apparatus comprising a central part (6 × 6 cm), two opposing open arms (40 × 6 cm), and two opposing closed arms (40 × 6 × 15 cm). The apparatus was raised 40 cm above the floor. Mice were placed in the center of the apparatus facing the closed arm and allowed to explore the maze for 5 min freely. Time spent and distance traveled in the center, open arms, and closed arms were measured using a video tracking system. For the acoustic startle response test, a startle box system (Panlab, Cornellà de Llobregat, Spain) containing a sound generator and accelerometer was used to record the amplitude of each mouse’s startle response. Mice were placed into a plexiglass cylinder and set on a weight-transducing platform in the sound-attenuating chamber. A white background noise (70 dB) was generated throughout the experiment. The acoustic startle response test began with 5 min of acclimatization. Mice were randomly exposed to startle stimuli (from 60 to 120 dB, 10-dB increments, 40-ms duration) with a variable (10–20 s) inter-stimulus interval to avoid habituation. Each sound intensity was presented six times. The peak amplitude of the startle response was measured.

### 4.4. Kinexus Protein Microarray

Brains from WT and *TauT*^−/−^ mice were cut into small pieces and rinsed in ice-cold phosphate-buffered saline to remove the blood and other contamination on the surface. Next, brains were wrapped in tinfoil and snap-frozen in liquid nitrogen. Subsequently, brains were shipped in dry ice to Kinexus Bioinformatics Corporation (Vancouver, BC, Canada) for Kinexus KAM-880 Antibody Microarray, which includes 518 pan-specific (for expression levels of these phosphoproteins) and 359 phosphorylation-site-specific antibodies (for phosphorylation). Briefly, 50 g of cell lysate from each sample was covalently labeled with a fluorescent dye. Free dye molecules were then removed at the completion of labeling reactions by gel filtration. After blocking non-specific binding sites, an incubation chamber was mounted onto the microarray to permit the loading of WT and *TauT*^−/−^ samples side by side on the same chip and to prevent mixing of the samples. Following sample incubation, unbound proteins were washed away. Each array produced a pair of 16-bit images, which were captured with a Perkin-Elmer ScanArray Reader laser array scanner (Waltham, MA, USA). Signal quantification was performed with ImaGene 8.0 from BioDiscovery (El Segundo, CA, USA), using the predetermined settings for spot segmentation and background correction. The resultant changes in spot intensity are expressed as the percentages of change for *TauT*^−/−^ mice compared with WT mice [% change from control (% CFC) = [(globally normalized signal intensity of *TauT*^−/−^ sample /globally normalized signal intensity of WT sample) × 100] − 100]. Changes of 50% CFC were considered significant changes. Globally normalized signal intensity is the background-corrected intensity value.

### 4.5. Statistical Analysis

Data are presented as mean ± standard error of the mean (SEM). Comparisons between the two groups were made using the Mann–Whitney test. Multiple comparisons were made using Kruskal–Wallis one-way ANOVA followed by Dunn’s multiple comparison test. Statistical significance was defined as *p* < 0.05. All statistical analyses were performed using GraphPad Prism (version 9.2.0; GraphPad Software, San Diego, CA, USA).

## Figures and Tables

**Table 1:** Startle response test. Numbers of mice showing a startle response to the startle pulse.

Gene Type	No. of Mice Showed Startle Response (%)						
	60 dB	70 dB	80 dB	90 dB	100 dB	110 dB	120 dB
WT	0/0(0%)	0/0(0%)	0/0(0%)	3/6(50%)	6/6(100%)	6/6(100%)	6/6(100%)
*TauT* ^−/−^	0/0(0%)	0/0(0%)	0/0(0%)	0/0(0%)	0/0(0%)	0/0(0%)	0/0(0%)

## Data Availability

Data are contained within the article.

## Supplementary Materials

The following supporting information can be downloaded at: https://www.mdpi.com/article/10.3390/metabo12070631/s1, Table S1: Effects of taurine depletion on protein expression and phosphorylation by protein microarray analysis.
