# Supplementary material for: Effects of Taurine Depletion on Body Weight and Mouse Behavior during Development
Source: Metabolites. 2022 Jul 9;12(7):631. doi: 10.3390/metabo12070631 (PMC9318136; doi:10.3390/metabo12070631)
Supplement: Supplementary file 1 [file metabolites-12-00631-s001.zip › metabolites-1716444-supplementary.pdf]

Table S1. Effects of taurine depletion on protein expression and phosphorylation by protein microarray analysis.

Target Protein Name	Phospho Site (Human)	Full Target Protein Name	%CFC
STAT3	Y705	Signal transducer and activator of transcription 3	9449
MKK6	Pan-specific	MAPK/ERK protein-serine kinase 6 (MKK6)	116
GRK2	Pan-specific	G protein-coupled receptor-serine kinase 2	109
PAK4	S474	p21-activated kinase 4	101
FRK	Y387	Tyrosine-protein kinase FRK	97
JAK3	Pan-specific	Janus protein-tyrosine kinase 3	91
Hsp47	Pan-specific	Heat shock 47 kDa protein (collagen-binding protein 1, colligin 1)	82
Snk	Pan-specific	Polo-like protein kinase 2 (serum -inducible kinase (SNK))	77
EGFR	Pan-specific	Epidermal growth factor receptor-tyrosine kinase	75
PRK2	Pan-specific	Protein kinase C-related protein-serine kinase 2	73
DUSP9	Pan-specific	Dual specificity protein phosphatase 9	73
SOCS2	Pan-specific	Suppressor of cytokine signaling 2	65
IkBa	Pan-specific	Inhibitor of NF-kappa-B alpha (MAD3)	63
STAT2	Pan-specific	Signal transducer and activator of transcription 2	61
IkBb	Pan-specific	Inhibitor of NF-kappa-B beta (thyroid receptor interacting protein 9)	57
MEK3	Pan-specific	MAPK/ERK protein-serine kinase 3 beta isoform (MKK3 beta)	55
Catenin b	Pan-specific	Catenin (cadherin-associated protein) beta 1	55
Cdc2 p34	Pan-specific	Cyclin-dependent protein-serine kinase 1	55
ErbB2	Pan-specific	ErbB2 (Neu) receptor-tyrosine kinase	54
JAK3	Y980+Y981	Janus protein-tyrosine kinase 3	51
A-Raf	Pan-specific	A-Raf proto-oncogene serine/threonine-protein kinase	51
NFkappaB p65	Pan-specific	NF-kappa-B p65 nuclear transcription factor	49
SODD	Pan-specific	Silencer of death domains (Bcl2 associated athanogene 4 (BAG4))	56
